# Globalising strategies to meet global challenges: the case of ageing and dementia

**DOI:** 10.7189/jogh.09.020310

**Published:** 2019-12

**Authors:** Mario A Parra, Stephen Butler, William J. McGeown, Louise A Brown Nicholls, David J Robertson

**Affiliations:** 1 School of Psychological Sciences and Health, University of Strathclyde, Glasgow, UK; 2 Alzheimer’s Scotland Dementia Research Centre, Edinburgh University, UK; 3 Autonomous University of the Caribbean, Barranquilla, Colombia; * Equal contribution, sorted alphabetically by surname

Dementia has been declared a Global Challenge [1]. However, strategies to tackle it are far from global. Epidemiological forecasts are more alarming for low and middle-income countries (LMIC) than for high-income countries (HIC), and yet provisions to support the former are scarce and, in some cases, as we discuss below, impractical. New initiatives are emerging to close these gaps. The Latin America and Caribbean Consortium on Dementia (LAC-CD) [2] and the Global Dementia Prevention Program (GloDePP Consortium; Wang, H. from Peking University and Chan, K.Y. from University of Edinburgh. Preventing dementia and improving dementia care: setting and addressing research priorities in China. Supported by Global Challenges Research Fund Networking Grants) are two examples (see **Figure 1**). They are seeking strategies to meet and map local and global challenges. Both consortia agree that actions to improve diagnosis and post-diagnostic support are of utmost priority. Here we discuss theory-driven, culturally valid, and interdisciplinary approaches that can yield affordable, reliable, and practical solutions to meet these outstanding needs.

**Figure 1:**
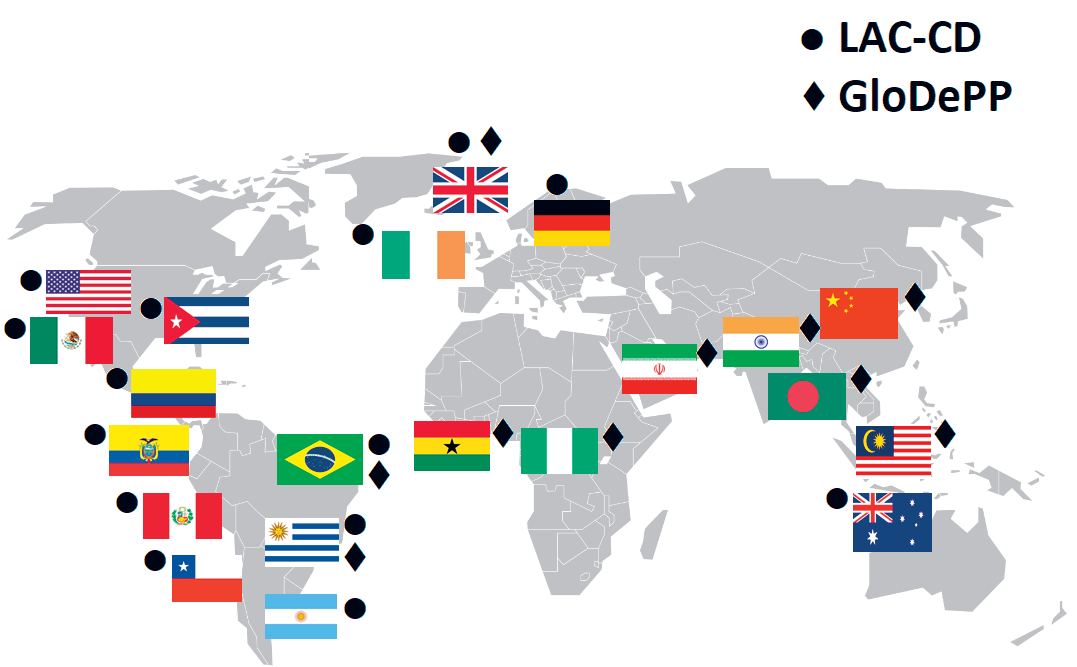
The Latin America and Caribbean Consortium on Dementia (LAC-CD) and the Global Dementia Prevention Program (GloDePP Consortium) are two emerging initiatives which hold potential to bridge LMIC and HIC across the globe, to globalise dementia strategies.

## Cognitive ageing in LMIC

How to manage and treat our ageing population is of critical concern to governments and healthcare providers across the world. Globally, due to declining fertility rates and increasing life expectancies, we are experiencing rapid population ageing. Within developing countries, age-related mortality is often viewed within the context of poverty, disease, and conflict, with little focus on the growing rates of dementia that rise in line with a growing population of older adults [3]. In 2015, Alzheimer’s Disease International (ADI) reported that there are over 9.9 million new cases of dementia each year worldwide, implying one new case every 3.2 seconds [4]. Investigations of the incidence rates in HIC have yielded a number of encouraging results. For example, in the UK, the Medical Research Council Cognitive Function and Ageing Studies I and II suggest the incidence rate for dementia has fallen by 20% over 20 years (with this mostly occurring in men) [5]. Likewise, in the USA – Framingham Heart study – incidence rates are reported to have dropped by a rate of approximately 20% per decade [6]. These decreases may be linked to factors such as those associated with reduced vascular risk [7]. In LMIC, despite reports that the incidence rate is 10% lower than in HICs, the risk profile is shifting in the opposite direction, with LMIC tending to exhibit increasing cardiovascular risk, suggesting that incidence rates might increase in these countries [4]. As the incidence and average duration of a disease determine its prevalence, globally the number of people living with dementia is increasing. ADI reported [8] that 46.8 million people worldwide live with dementia, and that this number will almost double every 20 years, to 74.7 million in 2030 and 131.5 million in 2050. They suggested that such an increase in prevalence would be attributable to increases in the numbers of people with dementia in LMIC. In 2015, 58% of all people with dementia lived in LMIC, and this is expected to rise to 63% in 2030 and 68% in 2050. Parra et al. [2] have recently identified factors linked to such an increase, among which, age, education, and changes in health care infrastructure were highlighted. However, more and better designed epidemiological studies are needed to identify the sources of such increases which, at present and for many countries, are still estimates from prediction models supported by the limited data available [2,4]. These countries must now adapt to ageing populations more quickly, and with comparatively fewer resources, than countries whose demographic ageing began much earlier.

Cognitive impairment is among the most feared aspects of ageing, and is a legitimate age-related health concern, potentially affecting wellbeing and independence. Crucially, individual differences play a role, and high cognitive ‘reserve’ is associated with greater resilience to the symptomology associated with Alzheimer’s disease (AD) [9]. Factors such as educational and occupational attainment are related to enhanced reserve, in terms of brain and cognitive functional capacity. Hypothetically, those with high reserve will tolerate pathology for longer, before reaching clinical levels of functioning. They would also exhibit reduced clinical severity, especially at the mild pathology stage, at which they may appear clinically normal. It has been recently reported that the education-dementia link in LMIC such as Latin American Countries is strong [10] and that although the mechanisms underpinning such a link are unclear, the concept of cerebral and cognitive reserve [9] offers a plausible explanation. Later in this opinion piece we shall address how such factors underpin various challenges faced by LMIC. For instance, mediators of cognitive reserve such as intellectual and lifestyle factors, can modify the course of ageing and render the task of separating normal and abnormal ageing trajectories an arduous one. We next discuss how interactions among such factors affect assessment of cognition late in life.

## CULTURALLY VALID ASSESSMENTS

**Figure:**
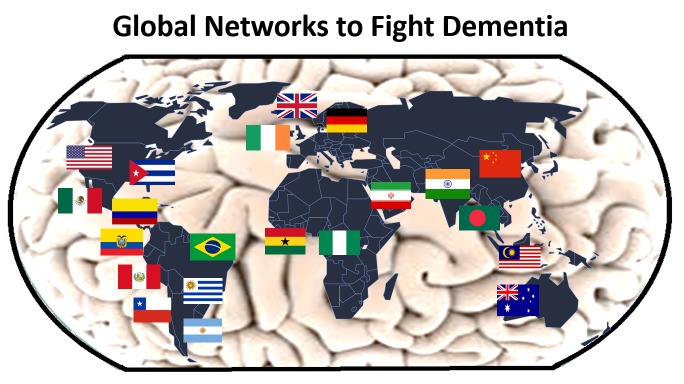
Photo: New initiatives are emerging which bring together countries from the Global South and Global North to identify shared barriers and to harmonise strategies that can help fight the dementia challenge globally (from the collection of Mario A Parra, used with permission).

Despite the gradual shift towards biologically oriented approaches to document the presence of AD [11], diagnostic criteria for dementia require evidence of cognitive decline which can be provided only via formal psychometric testing. Developing reliable neuropsychological tests to aid the diagnosis of dementia has proved a difficult task. From independent meetings recently held by LAC-CD and GloDePP, it has been acknowledged that the challenges experienced by countries across the globe are very similar (**Table 1**). Heterogeneity of assessment procedures (ie, clinical, neuropsychological, functional scales), lack of standardization, and sensitivity to demographic factors (ie, education, literacy, cultural idiosyncrasies, and environment) are shared barriers which need urgent action. For effective utilisation globally, optimal cognitive markers of AD should be highly sensitive and specific to its pathology, whereas they should be unaffected by healthy ageing, other types of dementia pathology, and the cultural background and literacy levels of those assessed. A new test, namely the Short-Term Memory Binding Test (STMBT; [21]), has been recently recommended by international consensus as it meets these criteria [22]. What features grant the STMBT such psychometric properties? The STMBT is a change detection task that presents stimuli that hold neither verbal properties nor long-term memory representations. The test taxes low level visual functions which draw little support from literacy or education. The test relies on a simple set of instructions (ie, detect differences across two consecutive visual arrays), thereby limiting any challenge for people with little formal education. The STMBT is not affected by prior knowledge, experience, or practice effects. These properties may hold the key to addressing long-standing limitations of neuropsychological tests.

**Table 1:** Mapping challenges to solutions, barriers and strategies to bridge local to global actions to empower low- and middle-income countries (LMIC) in the fight against dementia.

Challenges	Solutions	Barriers	Strategies
Culturally valid assessments	• To develop theory-driven cognitive tests to discriminate between normal ageing, non-progressive cognitive impairment, other types of dementia, or depression [12]. • To rely on bespoke rather than off-the-shelf assessments. • To avoid verbal tests which rely on literacy. • To tax low level functions (process pure) rather than complex multidimensional abilities. • To consider relevant cognitive constructs within cultural and behavioural contexts [12,13]. • To validate culturally unbiased tests across a range of equivalent tests currently available.	• Shared understanding of theory-driven assessments (ie, cognitive constructs underpinning these assessments). • Interactions between ageing, culture, and environment (eg, ethnographic factors underpinning stigma and social barriers). • Training of healthcare providers. • Cultural heterogeneity between and within countries (ie, ethnic minorities, stigma). • Lack of shared platforms for data collection, sharing, and big data analysis.	• To set up worldwide initiatives to raise awareness of challenges shared across LMIC (eg, linking LAC-CD & GloDePP). • To work with Diversity and Disparity Initiatives to explore and promote strategies that capture the heterogenous features that preclude standardization of assessments [13]. • To set up communication forums (eg, websites, workshops) to enable interactions, share practices, and encourage collaboration. • To develop integrated data collection, analysis, and sharing platforms.
Providing evidence of brain pathology	• To combine cognitive markers and low-cost technologies that can collect biological data (eg, eye-tracking) [14]. • The STMBT appears promising, and when combined with EEG, accuracy of discrimination should further increase [15]. • Data upload by LMIC clinics for cloud-based automated analysis and production of feedback reports. • Data-driven analyses have the potential to yield novel blood-based biomarkers.	• Training of health care providers and community workers to use novel technologies for dementia. • Identification of optimal set ups for portable assessments (eg, EEG, eye-tracking). • Determining how paradigm delivery could be uniform/controlled across assessment sites. • Large sample sizes are required for the data-driven development of blood-based biomarkers. • Blood-based biomarkers should be developed by training, testing and validating classifier models on diverse populations.	• Interdisciplinary efforts from computer sciences and biomedical engineering is opening new opportunities to implement robust methods such as machine learning algorithms towards data reduction, enhanced classification and diagnosis, and effective analytic pipelines for EEG data [16]. • Behavioural analytics will support the development of potential oculomotor biomarkers for dementia. • Large scale longitudinal research, acquiring a range of oculomotor metrics through tablets and phones, and alongside more established testing regimes, has the potential through machine learning to revolutionise both the diagnosis and the management of dementia. • Exploit data-sharing initiatives and biorepositories to acquire sufficient data for classifier development. • Develop low-cost technologies for point-of-care blood sampling and analytics. • Explore routes for scalable delivery of low-cost peripheral biomarkers for dementia. • Capitalise on emerging initiatives aimed at supporting the development of peripheral biomarkers for dementia [17].
Affordable interventions for dementia	• Lifestyle interventions that could help increase ‘healthy life expectancy’ across the globe [18]. • Use of technology (eg, VR/AR) to slow decline, restore functions, and prolong independent living [19,20].	• Limited awareness about the benefits of non-pharmacological interventions and healthy lifestyles. • Cultural, socio-economic, and ethical barriers which may deter patients and family members from using technology for intervention purposes [19,20].	• Use of information technologies to foster a cultural move towards healthier lifestyles [19]. • Interventions for dementia relying on VR/AR platforms should: i) be available for home use, reducing the financial/time pressure on users and local healthcare providers. ii) Ensure that cloud-based tutorials are developed to enable care-giver training and safe use of technology remotely. • Ethnographic studies to unveil barriers to implement technological development in LMIC.

Informant-based interviews (eg, AD8; [23]) are proving useful screening tools and are being validated in LMIC. However, awareness and stigma are barriers that may undermine their usefulness in such countries. There is consensus that proxy measurements should be complementary to the direct assessment of patients. This is even more relevant if we consider that informant-based interviews discriminate between nondemented individuals and those with cognitive impairment regardless of the aetiology. There is a need for assessment tools that are both sensitive and specific. For example, the STMBT suggested above holds both high sensitivity and specificity for AD [21,24,25]. Therefore, brief informant-based interviews and objective assessment such as the STMBT can be combined in LMIC settings to investigate their complementary value.

Why are cultural factors so relevant? A neuropsychological assessment for clinical purposes may be considered to have cross-cultural bias if there are significant cultural or language differences between the examiner, examinee, informants, tests, and/or social contexts. Most of the neuropsychological tests available in LMIC meet several of these criteria (see [2]). Assessment tools available to LMIC have been adapted from developed countries, and although properly translated, they do not account for cultural variation. Similar cognitive constructs may show different sensitivity to sociodemographic factors. For example, the STMBT and the Memory Binding Test (MBT) (see [22]) assess binding functions seemingly affected in the preclinical stages of dementia. Contrary to the STMBT, the MBT relies on free and cued selective reminding procedures, presents verbal materials which hold long-term memory representations, taxes associative memory functions which heavily rely on literacy and education, and is affected by prior knowledge and repetition effects. The former but not the latter test has proved insensitive to the level of education of the assessed individual. LMIC will need tests that circumvent the effects of such sociodemographic factors.

## PROVIDING EVIDENCE OF BRAIN PATHOLOGY

The new lexicon to define AD entails not only the documentation of cognitive impairment but of neuropathology. Within this new context, preclinical detection would require biomarker-based decisions and biomarker-focused computer-based algorithms for phenotyping and building risk profiles.

Biomarkers may be used to increase the certainty of diagnosis of AD. The recent A-T-N framework [11] emphasizes the hallmark features of AD, such as deposition of amyloid-beta (A) and changes in tau (T), and their identification with positron emission tomography or lumbar puncture. Accumulation of these abnormal proteins is associated with neurodegeneration (N) which normally triggers the clinical phase of the disease. These biomarkers are not frequently used in clinical practice, and given the cost, specialized equipment, invasiveness, and required clinical expertise (for data collection and analysis), they are unlikely to be widely available any time soon [8]. Complying with this emerging framework and attempting to adopt these biomarker methodologies presents LMIC with a set of important challenges. To overcome these, LMIC may instead turn towards novel proxy-biomarkers to improve confidence in diagnosis. It may be possible to develop high accuracy blood-based biomarkers for the detection of AD (that are minimally invasive and low-cost). In addition to targeted proteomics and metabolomics, data-driven methods may provide novel biomarkers which can rely on machine learning classifiers. For example, spectroscopic methods that provide biochemical profiles of blood serum/plasma, with subsequent classification using techniques such as artificial neural networks, have provided encouraging preliminary results for both sensitivity and specificity, albeit with small sample sizes (eg, [26]).

Electroencephalography (EEG) systems may also be used to enhance certainty of diagnosis. EEG is non-invasive, non-costly, widely available, and requires minimal clinical training. It offers an alternative biomarker, one that provides key information on the integrity of synaptic functioning, the disruption of which has also been detected very early in the disease course [27]. EEG biomarkers may focus on event-related potentials or resting state recordings, and on measures such as amplitude, coherence, synchronization likelihood, entropy, graph theory, phase-lag index, and Lempel-Ziv complexity (see [28] for a review). Progress is also being made in validating EEG biomarkers against other more standard markers of disease (eg, [29]). Standard EEG systems may already be available in hospitals/clinics in LMICs or, failing that, mobile EEG systems may be purchased at low cost. Mobile EEG may provide a promising avenue for assessment as the equipment is cheap to run, fast to set-up, can be transferred easily amongst settings, and can be applied with minimal training for the operator [30].

Recent evidence suggests that oculomotor behaviours linked to cognitive performance can yield additional biomarkers of AD (**Table 1**). Several potential oculomotor biomarkers of the disease have been identified in recent decades. Established deficits have been seen in higher level cognitive activities such as our ability to smoothly pursue a visual target, or our ability to inhibit a saccade and volitionally generate an antisaccade, in the oculomotor behaviour of reading and in low level physiological characteristics including pupil diameter, blink rate, and the speed of reflexive saccades [31]. The analysis of gazing and pupil responses during STMB performance significantly predicts the presence of AD pathology [32]. Gazing and pupil behaviours rest on a network that involves cortical and subcortical structures starting from the brainstem. It has been suggested that synaptic and circuit-level disruptions of subcortical systems controlling oculomotor responses are an early feature of AD which can potentially lead to amyloid accumulation and to the progression of neurodegeneration. Hence, the information drawn from eye movement behaviours linked to cognitive markers holds value as a potential biomarker for the disease.

As disease-modifying pharmacological treatments are not yet available, there is growing concern as to whether and how biomarker evidence would guide strategies to tackle this common disorder. We next address issues related to promising non-pharmacological interventions.

## AFFORDABLE INTERVENTIONS FOR DEMENTIA

As both genetic and lifestyle factors can account for variation in age-related cognitive change, there is clear opportunity to develop lifestyle interventions that could help increase ‘healthy life expectancy’ across the globe. For example, cognitive and physical activity levels as well as healthy diets, throughout the lifespan, are believed to help compensate for declines in cognitive and brain functioning (‘plasticity’). Indeed, observational studies have highlighted positive relationships between cognitive stimulation and cognitive outcomes. Additionally, “real-world” research is vitally important, both for informing future policy and practice, and for developing accessible, attractive intervention programmes that are feasible to implement on a large scale. It is also possible for community-based interventions, such as intergenerational engagement programmes, to bring wider societal benefits. In the area of lifestyle interventions, the use of technological developments is also opening new windows of opportunity (**Table 1**).

Prince et al. [33] looked at packages of care for dementia in LMIC. The review of the status quo of non-pharmacological approaches in HIC and LMIC confirms the need of more studies across such countries. The LAC-CD is currently drafting a knowledge-to-action framework which incorporates a Non-Pharmacological Intervention programme. This programme aims to introduce approaches which might prove feasible in the region due to social, cultural, idiosyncratic, and environmental factors. Examples are cognitive therapy, art therapy, musicotherapy, touch, animal assisted therapy, exercise, horticultural therapy, information and communication technologies interventions (computerized training, virtual reality and robotics), cognitive tele-rehabilitation, emotional intervention, and sensory stimulation. Experts from the region agreed that access to internet and ICT is rapidly growing in LAC. Hence, this would create opportunities to capitalise on emerging technologies to introduce these forms of intervention and validate them.

Dementia is particularly damaging to an individual’s independence and overall quality of life (eg, as a result of loss of memory and optimal judgement). However, it also affects those around the patient, who must take on the role of care giver, as patients’ personalities are slowly eroded, and whose mood and behaviour can become erratic. In light of this, a greater focus and more targeted interventions are required to ensure that those who develop dementia, and their caregivers, are provided with modern and cost-effective tools to manage this disease. In developed nations, the advent of virtual/augmented reality (VR/AR) technology is receiving greater attention, as a means of treating and managing a range of psychological/psychobiological disorders. In the case of dementia, the use of VR/AR is in its infancy [34]. However, there seems to be consensus that such technologies can help mitigate behavioural and psychological symptoms of patients having MCI and early-stage AD, better addressing their specific rehabilitation needs, as well as their caregivers’ intervention requirements [35,36]. This is particularly the case as the sophistication of the equipment increases along with a year-on-year reduction in cost (ie, related technology is now available via smart phones). In light of these advances in, and application of, VR/AR technologies, governments and healthcare providers should now look at the benefits that such an approach could bring to the management and treatment of dementia in LMIC.

In **Table 1** we have presented a list of solutions which can pave the way towards global strategies to tackle the dementia challenge. However, there still remain significant barriers to the implementation of such strategies. Among these, stigma and socio-cultural factors in LMIC are worth highlighting. The ADI’s 2012 report [37] reveals that stigma and social exclusion are major barriers for people with dementia and their caregivers. ADI reported that 24% of people with dementia and more than one in ten carers (11%) hide or conceal the diagnosis. Patients under the age of 65 fear facing issues in their workplace or children’s school. Social exclusion was identified in 40% of people with dementia with nearly 60% reporting that friends are the most likely people to avoid them followed by family members. To overcome such barriers, a significant amount of work with relevant stakeholders is needed. An emerging approach that could help bridge these gaps is Implementation Science [38]. The strategies here proposed are well aligned with factors that determine the implementation success. The solutions proposed in **Table 1** are cost-effective, relatively easy to implement, and can be flexibly adapted to the heterogenous socio-cultural contexts found in LMIC. More awareness is needed among relevant stakeholders and the general public [37]. Governments need to support the development of national dementia plans which health care workers, policymakers, and patients could adopt, carry out, and benefit from. Researchers from LMIC need to interact with such stakeholders to better understand the knowledge gaps and find answers to the challenges facing these groups. As Prince et al. [33] pointed out, more work is needed to assess whether packages of care for dementia developed in HIC can be implemented in LMIC. The strategies here proposed provide cues towards implementation actions. They are aimed at fostering collaborations between HIC and LMIC and it will be through such collaborations that these outstanding needs will be more rapidly addressed. We have seen progress over the last decade. Culture-free assessment methods developed in HIC [21] are now widely used in LMIC (eg, South America, [39]). A shared understanding of regional barriers that prompt urgent action is being fomented among relevant stakeholders of LAC-CD (see [2]). The model developed by this consortium could be generalised, and Implementation Science can provide the framework to help build such bridges.

## CONCLUSION

This opinion piece has highlighted strategies that can bridge gaps in LMIC. More work is needed to incorporate promising cognitive constructs (eg, memory binding) into culturally valid tests that can help separate normal from abnormal ageing trajectories. Such novel forms of cognitive assessment can be combined with affordable technologies to provide biomarker evidence of brain pathology which may further increase sensitivity and specificity. Cognitive biomarkers relying on these methodologies can be made available across health care levels, particularly in primary care for use in the community. By combining interventions that promote healthy lifestyles and novel technologies that empower patients and caregivers, it will be possible to foster cognitive reserves, slow cognitive decline, and prolong independent living. Collaborations that integrate international and interdisciplinary efforts will accelerate the delivery of more global solutions for tackling the dementia challenge.

## References

[1] Ferri C, Jackson J, Prince M. The global challenge of dementia. In: Ames D, Nurns A, O’Brien J (ed) Dementia, 4th edition. London: Hodder; 2010.

[2] Parra MA Baez S Allegri R Nitrini R Lopera F Slachevsky A Dementia in Latin America: Assessing the present and envisioning the future. Neurology. 2018;90:222-31. 10.1212/WNL.0000000000004897 29305437 PMC5791795

[3] Shaji KS Dementia care in developing countries: The road ahead. Indian J Psychiatry. 2009;51(Suppl1):S5 S7 21416017 PMC3038540

[4] Alzheimer’s Disease International. World Alzheimer Report 2015. The Global Impact of Dementia. An analysis of prevalence, incidence, cost & trends. Available: http://www.alz.co.uk/research/world-report-2015. Accessed: 6 December 2018.

[5] Matthews FE Stephan BC Robinson L Jagger C Barnes LE Arthur A Cognitive Function and Ageing Studies (CFAS) Collaboration A two decade dementia incidence comparison from the Cognitive Function and Ageing Studies I and II. Nat Commun. 2016;7:11398. 10.1038/ncomms11398 27092707 PMC4838896

[6] Satizabal CL Beiser AS Chouraki V Chêne G Dufouil C Seshadri S Incidence of Dementia over Three Decades in the Framingham Heart Study. N Engl J Med. 2016;374:523-32. 10.1056/NEJMoa1504327 26863354 PMC4943081

[7] Barnes DE Yaffe K The projected effect of risk factor reduction on Alzheimer’s disease prevalence. Lancet Neurol. 2011;10:819-28. 10.1016/S1474-4422(11)70072-2 21775213 PMC3647614

[8] Alzheimer’s Disease International. The World Alzheimer Report 2018, The state of the art of dementia research: New frontiers. Available: http://www.alz.co.uk/research/world-report-2018. Accessed: 6 December 2018.

[9] Stern Y Cognitive reserve in ageing and Alzheimer’s disease. Lancet Neurol. 2012;11:1006-12. 10.1016/S1474-4422(12)70191-6 23079557 PMC3507991

[10] Custodio N Wheelock A Thumala D Slachevsky A Dementia in Latin America: Epidemiological evidence and implications for public policy. Front Aging Neurosci. 2017;9:221. 10.3389/fnagi.2017.00221 28751861 PMC5508025

[11] Jack CR Bennett DA Blennow K Carrillo MC Feldman HH Frisoni GB A/T/N: An unbiased descriptive classification scheme for Alzheimer disease biomarkers. Neurology. 2016;87:539-47. 10.1212/WNL.0000000000002923 27371494 PMC4970664

[12] Logie RH Parra MA Della Sala S From cognitive science to dementia assessment. Policy Insights Behav Brain Sci. 2015;2:81-91. 10.1177/2372732215601370

[13] Babulal GM Quiroz YT Albensi BC Arenaza-Urquijo E Astell AJ Babiloni C Perspectives on ethnic and racial disparities in Alzheimer’s disease and related dementias: Update and areas of immediate need. Alzheimers Dement. 2019;15:292-312. 10.1016/j.jalz.2018.09.009 30555031 PMC6368893

[14] Fernandez G Orozco D Agamennoni O Schumacher M Sanudo S Biondi J visual processing during short-term memory binding in mild alzheimer’s disease. J Alzheimers Dis. 2018;63:185-94. 10.3233/JAD-170728 29614644

[15] Pietto M Parra MA Trujillo N Flores F Garcia AM Bustin J Behavioral and electrophysiological correlates of memory binding deficits in patients at different risk levels for alzheimer’s disease. J Alzheimers Dis. 2016;53:1325-40. 10.3233/JAD-160056 27372640

[16] Richiardi J, Achard S, Bunke H, Van De Ville D. Machine learning with brain graphs: predictive modeling approaches for functional imaging in systems neuroscience. Paper presented at the Signal Processing Magazine, IEEE. 2013.

[17] Khan TK Alkon DL Peripheral biomarkers of Alzheimer’s disease. J Alzheimers Dis. 2015;44:729-44. 10.3233/JAD-142262 25374110

[18] Kivipelto M Mangialasche F Ngandu T Lifestyle interventions to prevent cognitive impairment, dementia and Alzheimer disease. Nat Rev Neurol. 2018;14:653-66. 10.1038/s41582-018-0070-3 30291317

[19] Marasinghe KM Lapitan JM Ross A Assistive technologies for ageing populations in six low-income and middle-income countries: a systematic review. BMJ Innov. 2015;1:182-95. 10.1136/bmjinnov-2015-000065 26688747 PMC4680721

[20] Vaportzis E Martin M Gow AJ A Tablet for Healthy Ageing: The effect of a tablet computer training intervention on cognitive abilities in older adults. Am J Geriatr Psychiatry. 2017;25:841-51. 10.1016/j.jagp.2016.11.015 28082016 PMC5444526

[21] Parra MA Abrahams S Logie RH Della Sala S Visual short-term memory binding in Alzheimer’s disease and depression. J Neurol. 2010;257:1160-9. 10.1007/s00415-010-5484-9 20162428

[22] Costa A Bak T Caffarra P Caltagirone C Ceccaldi M Collette F The need for harmonisation and innovation of neuropsychological assessment in neurodegenerative dementias in Europe: consensus document of the Joint Program for Neurodegenerative Diseases Working Group. Alzheimers Res Ther. 2017;9:27. 10.1186/s13195-017-0254-x 28412978 PMC5392959

[23] Galvin JE Roe CM Powlishta KK Coats MA Muich SJ Grant E The AD8: a brief informant interview to detect dementia. Neurology. 2005;65:559-64. 10.1212/01.wnl.0000172958.95282.2a 16116116

[24] Cecchini MA Yassuda MS Bahia VS de Souza LC Guimaraes HC Caramelli P Recalling feature bindings differentiates Alzheimer’s disease from frontotemporal dementia. J Neurol. 2017;264:2162-9. 10.1007/s00415-017-8614-9 28894929

[25] Della Sala S Parra MA Fabi K Luzzi S Abrahams S Short-term memory binding is impaired in AD but not in non-AD dementias. Neuropsychologia. 2012;50:833-40. 10.1016/j.neuropsychologia.2012.01.018 22289292

[26] Ryzhikova E Kazakov O Halamkova L Celmins D Malone P Molho E Raman spectroscopy of blood serum for Alzheimer’s disease diagnostics: specificity relative to other types of dementia. J Biophotonics. 2015;8:584-96. 10.1002/jbio.201400060 25256347 PMC4575592

[27] Babiloni C Vecchio F Lizio R Ferri R Rodriguez G Marzano N Resting state cortical rhythms in mild cognitive impairment and Alzheimer’s disease: electroencephalographic evidence. J Alzheimers Dis. 2011;26 Suppl 3:201-14. 10.3233/JAD-2011-0051 21971461

[28] Dauwels J Vialatte F Cichocki A Diagnosis of Alzheimer’s disease from EEG Signals: Where are we standing? Curr Alzheimer Res. 2010;7:487-505. 10.2174/156720510792231720 20455865

[29] Maestú F Cuesta P Hasan O Fernandéz A Funke M Schulz PE The Importance of the Validation of M/EEG With Current Biomarkers in Alzheimer’s Disease. Front Hum Neurosci. 2019;13:1-10. 10.3389/fnhum.2019.00017 30774588 PMC6367251

[30] Cecchi M Moore DK Sadowsky CH Solomon PR Doraiswamy PM Smith CD A clinical trial to validate event-related potential markers of-Alzheimer’s-disease in outpatient settings. Alzheimers Dement (Amst). 2015;1:87-394. 10.1016/j.dadm.2015.08.004 27239520 PMC4879492

[31] Coubard OA What do we know about eye movements in Alzheimer’s disease? The past 37 years and future directions. Biomarkers Med. 2016;10:677-80. 10.2217/bmm-2016-0095 27348080

[32] Fernandez G Orozco D Agamennoni O Schumacher M Sanudo S Biondi J Visual processing during short-term memory binding in mild Alzheimer’s Disease. J Alzheimers Dis. 2018;63:185-94. 10.3233/JAD-170728 29614644

[33] Prince MJ Acosta D Castro-Costa E Jackson J Shaji KS Packages of care for dementia in low- and middle-income countries. PLoS Med. 2009;6:e1000176-1000176. 10.1371/journal.pmed.1000176 19888456 PMC2766257

[34] Parra MA Kaplan RI Predictors of performance in real and virtual scenarios across age. Exp Aging Res. 2019;45:180-98. 10.1080/0361073X.2019.1586106 30898079

[35] Doniger GM Beeri MS Bahar-Fuchs A Gottlieb A Tkachov A Kenan H Virtual reality-based cognitive-motor training for middle-aged adults at high Alzheimer’s disease risk: A randomized controlled trial. Alzheimers Dement (N Y). 2018;4:118-29. 10.1016/j.trci.2018.02.005 29955655 PMC6021455

[36] García-Betances RI Jiménez-Mixco V Arredondo MT Cabrera-Umpiérrez MF Using virtual reality for cognitive training of the elderly. Am J Alzheimers Dis Other Demen. 2015;30:49-54. 10.1177/1533317514545866 25107931 PMC10852905

[37] Alzheimer’s Disease International. World Alzheimer Report 2012: Overcoming the stigma of dementia. Available: https://www.alz.co.uk/research/world-report-2012. Accessed: 6 December 2018

[38] Bauer MS Damschroder L Hagedorn H Smith J Kilbourne AM An introduction to implementation science for the non-specialist. BMC Psychol. 2015;3:32-32. 10.1186/s40359-015-0089-9 26376626 PMC4573926

[39] Parra MA Overcoming barriers in cognitive assessment of Alzheimer’s disease. Dement Neuropsychol. 2014;8:95-8. 10.1590/S1980-57642014DN82000002 29213888 PMC5619114

